# Detection of Norovirus in Saliva Samples from Acute Gastroenteritis Cases and Asymptomatic Subjects: Association with Age and Higher Shedding in Stool

**DOI:** 10.3390/v12121369

**Published:** 2020-11-30

**Authors:** Eduard Anfruns-Estrada, Aurora Sabrià, Cristina Fuentes, Sara Sabaté, Efrén Razquin, Thais Cornejo, Rosa Bartolomé, Nuria Torner, Conchita Izquierdo, Nuria Soldevila, Lorena Coronas, Angela Dominguez, Rosa M. Pintó, Albert Bosch, Susana Guix

**Affiliations:** 1 Enteric Virus Laboratory, Department of Genetics, Microbiology and Statistics, University of Barcelona, 08028 Barcelona, Spain; eanfruns@ub.edu (E.A.-E.); aurorasabria@ub.edu (A.S.); cfuentes@ub.edu (C.F.); rpinto@ub.edu (R.M.P.); abosch@ub.edu (A.B.); 2 Nutrition and Food Safety Research Institute (INSA·UB), University of Barcelona, 08921 Barcelona, Spain; 3 Laboratori de l’Agència de Salut Pública de Barcelona, 08001 Barcelona, Spain; ssabate@aspb.cat (S.S.); erazquin@aspb.cat (E.R.); 4 Vall d’Hebron University Hospital, 08035 Barcelona, Spain; thais.cornejo@vhir.org (T.C.); rbartolome@vhebron.net (R.B.); 5 Department of Health, Generalitat of Catalonia, 08005 Barcelona, Spain; nuria.torner@gencat.cat (N.T.); conchita.izquierdo@gencat.cat (C.I.); 6 CIBER de Epidemiología y Salud Pública (CIBERESP), Instituto de Salud Carlos III, 28029 Madrid, Spain; nsoldevila@ub.edu (N.S.); coronas@ub.edu (L.C.); angela.dominguez@ub.edu (A.D.); 7 Department of Medicine, University of Barcelona, 08036 Barcelona, Spain

**Keywords:** human norovirus, saliva, acute gastroenteritis, PMAxx-viability RTqPCR, asymptomatic infection, FUT2 genotyping

## Abstract

Norovirus infections are a leading cause of acute gastroenteritis outbreaks worldwide and across all age groups, with two main genogroups (GI and GII) infecting humans. The aim of our study was to investigate the occurrence of norovirus in saliva samples from individuals involved in outbreaks of acute gastroenteritis in closed and semiclosed institutions, and its relationship with the virus strain, virus shedding in stool, the occurrence of symptoms, age, and the secretor status of the individual. Epidemiological and clinical information was gathered from norovirus outbreaks occurring in Catalonia, Spain during 2017–2018, and stool and saliva samples were collected from affected and exposed resident individuals and workers. A total of 347 saliva specimens from 25 outbreaks were analyzed. Further, 84% of individuals also provided a paired stool sample. For GII infections, norovirus was detected in 17.9% of saliva samples from symptomatic cases and 5.2% of asymptomatic individuals. Positivity in saliva occurred in both secretors and nonsecretors. None of the individuals infected by norovirus GI was positive for the virus in saliva. Saliva positivity did not correlate with any of the studied symptoms but did correlate with age ≥ 65 years old. Individuals who were positive in saliva showed higher levels of virus shedding in stool. Mean viral load in positive saliva was 3.16 ± 1.08 log_10_ genome copies/mL, and the predominance of encapsidated genomes was confirmed by propidium monoazide (PMA)xx-viability RTqPCR assay. The detection of norovirus in saliva raises the possibility of oral-to-oral norovirus transmission during the symptomatic phase and, although to a lesser extent, even in cases of asymptomatic infections.

## 1. Introduction

Human noroviruses (HuNoV) are associated with 18% of diarrheal diseases worldwide and cause 200,000 deaths among children every year, mostly in developing countries [1,2]. Infections are mainly transmitted through the fecal–oral route, often involving contaminated food and water, although transmission through vomit is also well documented [3,4,5]. HuNoV are classified into 10 genogroups, with genogroup I (GI) and genogroup II (GII) being the most prevalent affecting humans, with more than 9 and 27 genotypes within GI and GII, respectively [6]. Individual differences in susceptibility to infections by different genotypes have been described depending on the expression of histoblood group antigens (HBGA), the expression of which is primarily determined by the FUT2 gene [7]. Although secretor-negative individuals are resistant to several genotypes, infections in nonsecretors have been documented for GI.2, GI.3, GII.1, GII.2, GII.3, GII.6, GII.7, GII.4, and GII.17 [7,8,9].

HuNoV RNA has been detected by RTqPCR in saliva and the oral cavity of infected individuals with gastroenteritis, but the number of studies is very scarce. Kirby et al. [10] detected 24% HuNoV RNA positivity in mouthwashes from hospitalized patients with GII.4 gastroenteritis, with a slightly positive association with previous vomiting, especially during the 24 h before specimen collection. They also observed viral RNA positivity in early morning oral samples up to 10–15 days after disease onset in 6 individuals of a GII.3 family outbreak, despite some of them not having vomited. Detection of HuNoV RNA in saliva in the absence of vomiting has also been confirmed in an immunocompromised individual [11]. Finally, a screening of oral swabs from children with acute gastroenteritis identified RNA from diarrheal viruses, including HuNoV, in 16.9% of samples [12].

The main objectives of the current work were to investigate the occurrence of HuNoV in saliva samples from individuals involved in outbreaks of acute gastroenteritis in closed and semiclosed institutions and its relationship with the virus strain, the occurrence of symptoms and virus shedding in stool, and the secretor status of the individual. We also evaluated if saliva samples would be a suitable alternative specimen to stools to diagnose HuNoV infection. Finally, selected saliva specimens were analyzed using a viability RTqPCR assay to avoid detection of noninfectious viral particles and obtain a better assessment of infectivity [13,14,15].

## 2. Materials and Methods

### 2.1. Definitions and Sample Collection

HuNoV infection, acute gastroenteritis, and confirmed cases of HuNoV acute gastroenteritis were defined as previously described [16]. Epidemiological and clinical information was gathered from 25 HuNoV outbreaks occurring in closed and semiclosed institutions (nursing homes, socio-health centers, kindergartens, preschool centers, and others) in Catalonia, Spain during 2017–2018. Saliva specimens were collected from 347 subjects, including affected and exposed residents (*n* = 194) and workers (*n* = 148) (information could not be obtained for 5 subjects). Two hundred and ninety-two individuals (84%) provided paired stool and saliva samples, and 55 individuals (16%) only provided saliva. Further, 159 individuals also provided a stool specimen as part of the routine outbreak investigation. Finally, individuals were asked to provide 1–2 serial additional saliva and/or stool samples collected at approximately 10-day intervals; 38 subjects provided 1 additional paired stool and saliva, 84 individuals provided only serial stool samples, and 50 of them provided a second serial stool sample. This study was conducted in accordance with the Declaration of Helsinki and was approved by the Ethics Committee of the University of Barcelona (IRB00003099).

Saliva specimens were collected by asking the individuals to spit 2–3 mL of saliva directly into a sterile polypropylene test tube, preferably in the morning, before eating and before brushing teeth. For symptomatic individuals, mean time between onset of symptoms and first sample collection was 4 ± 3.4 days (0–20 days). All specimens were transported to the laboratory at 4 °C and further stored at −20 °C until testing.

### 2.2. HuNoV Testing

Presence of HuNoV in stool was assessed by real-time RTqPCR at the Hospital Universitari Vall d’Hebron and the Agència de Salut Pública de Barcelona (ASPB), as previously described [16]. Viral RNA was extracted from a 10% stool suspension using the NucliSENS^®^ easyMAG^®^ system (BioMérieux, Marcy-L’Etoile, France), and the presence of HuNoV was assessed by real-time RT-PCR according to ISO 15216-2:2019 [17]. Saliva samples were screened for HuNoV by RTqPCR at the Enteric Virus Laboratory (University of Barcelona). Viral RNA was extracted from 0.2–0.5 mL of saliva, and viral RNA was quantified according to the ISO 15216-1:2017 method [18]. Limit of quantification (LOQ) of viral RNA in saliva was determined at 3.23 and 2.95 log_10_ genome copies/mL for GI and GII, respectively.

### 2.3. Secretor Status Genotyping

Genotyping for FUT2 gene was performed by PCR amplification of nucleic acid extracted from saliva using primers described by Marionneau et al. [19], followed by Sanger sequencing. Analysis of the chromatogram peaks was used to determine homozygosity or heterozygosity for G428A mutation.

### 2.4. HuNoV Genotyping

Positive specimens were genotyped for polymerase and capsid genes by seminested RT-PCR [20]. RT-PCR products were purified and sequenced on an ABI Prism 3700 automatic sequencer (Applied Biosystems). Genotypes were assigned using the Norovirus Genotyping Tool [21].

### 2.5. HuNoV PMAxx-Viability RTqPCR

A propidium monoazide (PMA) viability RTqPCR assay was performed as previously described [22], with minor modifications. Briefly, saliva samples were incubated with 50 μM PMAxx (Biotinum) and 0.5% Triton X-100 in the dark at room temperature (RT) for 10 min at 150 rpm and exposed to light for 15 min using a photoactivation system (Led-Active Blue, Geniul). Nucleic acid extraction was performed using the NucliSens^®^ miniMAG magnetic kit (BioMérieux) according to the manufacturer’s instructions, and viral genomes were quantified according to the ISO 15216-1:2017 method [18]. An aliquot of saliva which had not been treated with PMAxx was extracted simultaneously.

### 2.6. Statistical Analysis

Chi-square tests were used to compare categorical variables and Student’s *t* test was used to compare continuous variables, using Open Source Epidemiologic Statistics for Public Health [23]. *p* values < 0.05 were considered statistically significant.

## 3. Results

### 3.1. Epidemiological Features of Studied Outbreaks

The main epidemiological features of 25 studied outbreaks are summarized in Table 1. Most outbreaks (84%) were person-to-person transmitted. Nine outbreaks were caused by GI strains, 14 by GII strains, and 2 outbreaks were caused by mixed genotypes belonging to GI and GII. Stool and/or saliva specimens, together with epidemiological data, were analyzed from 506 individuals, including 304 users attending the affected institution and 197 exposed workers, of which 78.0% and 36.0%, respectively, developed acute gastroenteritis symptoms and were considered as cases (Table 2). Detection of HuNoV in stool by RTqPCR was used to determine HuNoV infection status. HuNoV RNA was detected in 75.4% of stool specimens from symptomatic cases and 19.2% of asymptomatic exposed subjects, confirming the occurrence of asymptomatic infections. The percentage of symptomatic cases which were confirmed by detection of HuNoV in stool by RTqPCR was significantly higher in outbreaks associated with GII strains than GI strains (79.4% vs. 61.4%, *p* = 0.002).

**Figure 1:**
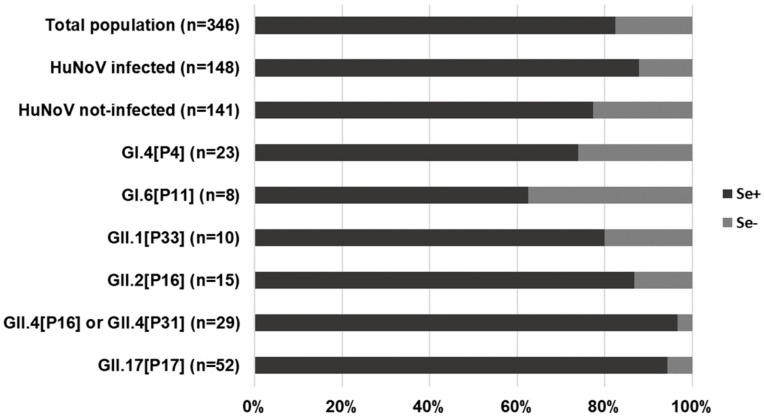
Distribution of secretor and nonsecretor subjects in different subgroups of studied individuals.

Secretor status was determined from 346 subjects by FUT2 genotyping, with an overall prevalence of secretors of 82.4%. While most HuNoV infections occurred among secretors, infection could also be confirmed by detection of viral shedding in stool for 18 nonsecretor individuals, related to outbreaks caused by GI.4[P4] (*n* = 6), GI.6[P11] (*n* = 3), GII.1[P33] (*n* = 2), GII.2[P16] (*n* = 2), GII.4[P31] (*n* = 1), GII.17[P17] (*n* = 3), and GI.3[P13] + GI.2 (*n* = 1). All infected nonsecretors, with the exception of an 88-year-old resident from a GII.17[P17] outbreak who did not report symptoms, met the clinical definition of acute gastroenteritis. The viral sequence from the stool of nonsecretor patients was confirmed in subjects infected by GI.4[P4] (*n* = 3), GI.6[P11] (*n* = 3), GII.2[P16] (*n* = 1), and GII.4[P31] (*n* = 1). Our data confirm that natural clinical infections by these genotypes may occur in nonsecretors. As compared with the overall percentage of nonsecretors among all infected subjects (12.2%), the percentage of nonsecretors among subjects infected by GI.4[P4] and GI.6[P11] was markedly higher, and for subjects infected by GII.4[P16] or GII.4[P33] and GII.17[P17], it was markedly lower (Figure 1), suggesting that the ability of GI.4[P4] and GI.6[P11] viruses to infect both secretors and nonsecretors is similar.

### 3.2. Occurrence of HuNoV RNA in Saliva Samples

**Figure 2:**
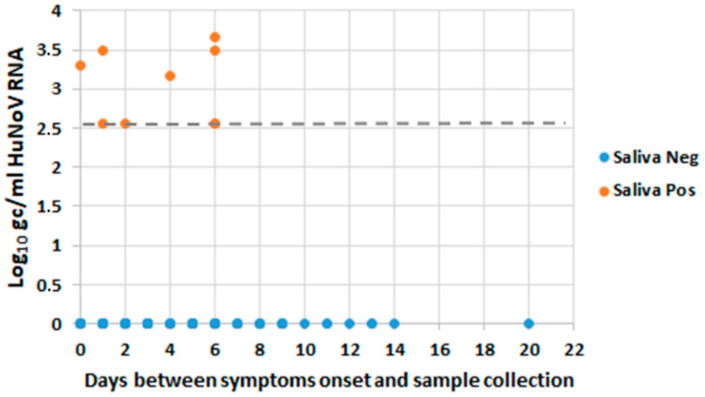
Individual HuNoV RNA titers according to time of sample collection for saliva-positive and saliva-negative subjects. Viral titers are expressed as log_10_ genome copies/mL of saliva. Dashed line indicates limit of quantification (LOQ) of viral RNA in saliva.

A total of 347 saliva samples were analyzed by RTqPCR. HuNoV RNA prevalence in saliva was significantly higher in symptomatic cases as compared with asymptomatic exposed subjects (12.1% vs. 3.6%; *p* = 0.001). Information regarding time from sample collection and onset of symptoms was available for 135 samples (123 saliva negative and 12 saliva positive). No significant differences were observed between time from sample collection and onset of symptoms between saliva-positive and saliva-negative groups (3.33 ± 2.39 vs. 4.11 ± 3.48 days, respectively; *p* = 0.135). Figure 2 shows individual HuNoV RNA titers according to days of sample collection. Of note, all positive saliva samples were only identified in subjects exposed to GII strains; saliva samples from outbreaks caused by GI genotypes were all negative (Table 3). When considering subjects associated with GII outbreaks only, HuNoV RNA was detected in 17.9% of symptomatic cases and in 5.2% of asymptomatic exposed subjects (*p* = 0.003). When comparing RTqPCR diagnosis in saliva to RTqPCR in stool as a reference method, stool samples remain preferable specimens for the diagnosis of HuNoV infections (sensitivity 11.5%, specificity 95.1%).

Of the 30 subjects who were positive in saliva, 83.3% reported to have symptoms. A paired stool sample was available for 23 of them. Of those, 17 were positive in both saliva and stool (73.9%), and 6 were only positive in saliva (26.1%). Only a resident and a worker from an outbreak occurring at a nursing home had detectable HuNoV RNA in saliva despite having a negative stool specimen and not reporting symptoms.

Mean HuNoV GII viral load in positive saliva samples was 3.16 ± 1.08 log_10_ genome copies/mL (this value was calculated taking into account that 60% of positive samples fell below the LOQ of the assay). Mean percentage for mengovirus recovery was 62.5 ± 36.5, and mean percentage of RTqPCR efficiency was 86.8 ± 20.0 and 80.2 ± 19.5 for GI and GII, respectively. No significant differences were observed in saliva viral load according to occurrence of symptoms or genotype. No association was observed between the days passed between sample collection and onset of symptoms. Due to the low viral concentration present in saliva, sequence agreement between viruses isolated in stool and saliva was attempted for three of the saliva samples with higher titers but could only be confirmed for two of them.

Examination of serial stool samples collected for subjects with HuNoV shedding in stool on the first specimen revealed that 71.1% of them (37 out of 52 individuals) remained positive after 11 ± 6.4 days from the first sample, and that 22.2% of the analyzed third specimens collected after 11 ± 3.4 days from the second sample (6 out of 27) still remained positive. In saliva, however, all 38 samples collected at 10 ± 5.2 days from the first sample were negative.

### 3.3. Analysis of Factors Associated with HuNoV RNA Saliva Positivity

Since occurrence of vomiting, especially during the 24 h before specimen collection, has been suggested to be associated with the occurrence of HuNoV RNA in saliva [10], we tested if this association was present, as well as with other clinical symptoms and host factors such as secretor status or age. Since we had not observed any positive saliva sample among subjects involved in GI outbreaks, we excluded data from GI outbreaks from this analysis (Table 4). Our data did not show vomiting as a significant risk factor for HuNoV positivity in saliva. Prevalence of HuNoV in saliva did not correlate with any other clinical symptom or with previous consumption of proton pump inhibitors, secretor status, or sex. Occurrence of nausea and absence of fever did slightly correlate with HuNoV RNA positivity in saliva, but differences were not statistically significant. On the contrary, age ≥ 65 years old was identified as a significant risk factor (*p* = 0.022). Analyses performed in all cases, including the ones involved in GI outbreaks, confirmed the same results (data not shown). Regarding the total duration of symptoms, no significant differences were observed between saliva-positive and saliva-negative cases (2.6 ± 1.8 vs. 2.8 ± 1.4 days, respectively; *p* = 0.118).

### 3.4. Occurrence of HuNoV RNA in Saliva and Levels of Shedding in Stool

**Figure 3:**
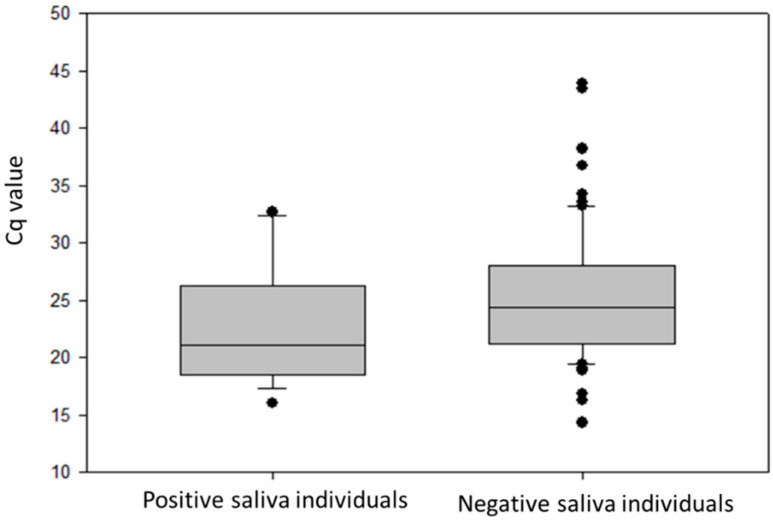
Viral load shed by saliva-positive and saliva-negative subjects under study, represented as box plots (median, quartiles (box), 95% range (whiskers), and outliers (circles)).

Viral load was examined in stool specimens of GII-infected individuals and compared between groups of cases which were positive or negative for viral RNA in saliva. Mean Cq values obtained during screening were used instead of viral titers (Figure 3). Mean Cq values were significantly higher in the group with positive saliva (22.45 ± 5.02 versus 24.89 ± 4.91, *p* = 0.02), suggesting that the overall level of viral replication in these individuals may be higher.

### 3.5. Analysis of HuNoV Capsid Integrity in Saliva Using a Viability PMA RTqPCR Assay

In order to examine whether HuNoV RNA was encapsidated and “potentially infectious”, a viability RTqPCR assay using propidium monoazide (PMA) was attempted on one saliva specimen with a high viral load from a subject infected by GII.P16_GII.2. A previous validation of the PMA RTqPCR assay was performed to confirm the suitability of this procedure to be applied to saliva by artificially spiking a negative saliva specimen with a 10% stool suspension and inactivating the virus by heating the saliva up to 95 °C for 5 min (Table 5). The use of PMA reduced the amplification of viral genomes in the heat-inactivated saliva by 0.97 log_10_, confirming a good performance. As measured by the PMA-viability assay, heat treatment of spiked saliva resulted in a viral titer decrease of >4.75 log_10_ (from 6.69 ± 0.01 to 1.94 ± 0.47 log_10_ genome copies/mL). In the naturally contaminated saliva, only a 0.3 log_10_ reduction was seen by PMA treatment, indicating that approximately 50% of genomes present in saliva were encapsidated within intact capsids and were “potentially infectious”.

## 4. Discussion

In our study, we detected HuNoV RNA in 12.1% of studied cases and 3.6% of asymptomatic exposed subjects. This overall prevalence is slightly lower than data reported by Kirby et al. [10], with differences being probably due in part to differences in the date of sample collection and to the fact that our study included cases infected by a higher diversity of genotypes. While all patients included in that previous study were infected by GII.4 or GII.3 strains [10], our study included outbreaks caused by at least nine different genotypes, including both GI and GII. In addition to cases infected by GII.4 strains, we also detected HuNoV RNA in saliva from GII.2 and GII.17 cases. No positive saliva samples were observed for GII.1[P33] cases, probably due in part to the lower number of samples tested for this particular genotype. Of note, we did not detect any positive samples in the saliva from subjects related to GI outbreaks. This finding could be related to different biological properties and/or host responses between GI and GII strains during infection, but differences between sensitivities of GI and GII RTqPCR assays used for screening could also partially explain it. Indeed, the proportion of cases with a negative RTqPCR result in stool was also significantly higher for GI than GII cases (38.6% vs. 20.6%, respectively), suggesting that performance of RTqPCR assays or viral load in stool could differ between the two genogroups. Evidence of the association between viral load and genogroup is limited, but some data indicate that shedding may be higher for GII genotypes [25,26,27].

An important observation of this study is that subjects with HuNoV occurrence in saliva also shed significantly higher viral loads in stool. This finding suggests that the overall level of viral replication in these individuals may be higher and could translate into differences in the ease of transmission. As highlighted by others [28], differences in viral replication do not clearly correlate with symptoms, which may be more related to the host immune and antiviral responses. In our study, we did not observe a correlation of occurrence of HuNoV in saliva and any of the studied symptoms (diarrhea, abdominal pain, nausea, fever, nausea, and vomiting), consistent with the idea that symptomatology is not associated with increased viral replication. What we did observe was a significant association with age, with individuals ≥65 years old showing a prevalence of HuNoV RNA in saliva of 27.3%, as compared with 11.1% in younger ones (Table 4). A higher initial viral load, as well as a longer duration of shedding, has been reported for older individuals [29], consistent with a likely higher replication and with the association of saliva positivity and age. In our study, we also found significantly lower mean Cq values in stool for GII-infected individuals ≥65 years old as compared with younger ones (23.42 ± 5.29 vs. 25.45 ± 5.35, *p* = 0.015), confirming that viral replication was likely higher in the elderly. Of note, this observation was not detected for GI-infected subjects. In addition to a higher level of viral replication, age has also been identified as a risk factor for gastroesophageal reflux disease [30], and it is likely that this could also facilitate the occurrence of HuNoV in saliva.

FUT2 genotyping allowed us to identify 18 nonsecretor subjects who had HuNoV shedding in stool, confirming their infection status, with presentation of acute gastroenteritis symptoms in all of them but one. While symptomatic infection of HuNoV in nonsecretors has been documented for GI.2, GI.3, GII.1, GII.2, GII.3, GII.6, GII.7, GII.4, and GII.17 genotypes [7,8,9], our report confirms for the first time infection by the GI.4 genotype as well, with sequence confirmation in stool. Binding of GI.4 viruses to carbohydrates from secretor-negative individuals has been documented in vitro [31], but to our knowledge, natural infections have not been reported yet. While GII.4 and GII.17 genotypes are thought to be secretor specific [32,33], our study identified one nonsecretor subject infected by GII.4 and three nonsecretor subjects involved in outbreaks caused GII.17. Despite that our data cannot completely rule out that these latter three subjects could have been infected by a different genotype, it is likely that they were infected by the same strain causing the outbreak. Indeed, cases of infections by GII.4 and GII.17 affecting nonsecretors have been documented previously [24,26,34].

The use of clinical specimens from the oral cavity (oral swabs, mouthwash, or saliva) has been assessed by some authors as a suitable alternative specimen to stools for the virological diagnosis of acute gastroenteritis due to its noninvasive nature and ease of collection. As reported by other authors [12], our data clearly support that saliva would not be a suitable specimen. Despite the fact that we were able to identify positive saliva in individuals who were negative in stool, saliva showed very poor sensitivity compared with stool. Noninvasive saliva testing has, nonetheless, been validated as a useful approach to confirm and rapidly screen HuNoV infection by detection of specific IgA or IgG antibodies [35,36].

Despite an association with vomiting prior to specimen collection having been documented [10,12], our data did not show an association between vomiting or nausea and higher prevalence of HuNoV RNA in saliva. However, in our study, saliva samples had been taken later than 24 h after the onset of symptoms (approximately 4 ± 3.4 days), and this could explain the lack of association. The study published by Zhou et al. [12] also acknowledged the occurrence of HuNoV GII as well as of other enteric viruses such as rotavirus, adenovirus, and astrovirus in oral swabs from children who presented with diarrhea only.

The mechanism behind detection of HuNoV in saliva when the patient does not show vomiting is still unknown. The presence of HuNoV in the absence of preceding vomiting could be explained by reflux of gastrointestinal contents to the oral cavity and binding of viruses to HBGA receptors expressed at the oral cavity, but a low level of viral replication in mucosal epithelial cells of the oral or pharyngeal cavity should not be ruled out. The small intestine is regarded as the primary site for HuNoV infection, and data on which specific cell types are targeted by the virus are just being revealed. Propagation of HuNoV in the human intestinal enteroid culture model indicates that enterocytes are a permissive target cell for viral replication [37], and in-depth histological examination of a pediatric intestinal transplant recipient presenting with severe HuNoV gastroenteritis identified markers of viral replication in specialized epithelial cells of the small intestine with sensory and endocrine functions [38]. Whether a low level of virus could be replicating in a small subset of specialized epithelial cells in the upper gastrointestinal tract and/or the pharynx in certain individuals has not been addressed. Of interest, two recent studies have detected diarrheal viruses, including HuNoV, in the nasopharynx of 11.4% [39] and 15.8% [40] of children with acute gastroenteritis, and a prevalence of 0.5% has also been reported for HuNoV RNA in respiratory secretions of children with influenza-like illness [41]. An independent study on subjects without acute gastroenteritis found HuNoV-specific memory B cells in tonsils [42], although they could not detect any marker of viral replication and they did not explore the origin or the role of these cells in that tissue.

Our data show that occurrence of HuNoV in saliva may not be a common finding to all infected individuals. Differences related to virus strain and host factors such as the occurrence of salivary immunoglobulins or a particular composition of the oral microbiota might explain why not all infected individuals showed HuNoV RNA in their saliva. However, despite not being a widespread phenomenon, the occurrence of HuNoV RNA in saliva opens the door to a possible oral–oral transmission. Although the RNA concentrations of the virus in saliva appear to be much lower than those present in feces, or vomit, given the low infectious dose described for HuNoV [8,43,44,45], concentrations could be high enough to infect a new host. In addition, using the PMA-RTqPCR viability assay we confirmed that saliva may contain a high proportion of viruses with intact capsids, thus being potentially infectious.

In conclusion, HuNoV may be associated with oral-to-oral transmission through saliva, contributing to the spread of infection. Further studies are needed to evaluate the epidemiological relevance of this transmission mode and to better understand host and viral factors favoring the occurrence of HuNoV in saliva.

## Figures and Tables

**Table 1:** Main epidemiological features of the 25 outbreaks used in the study.

Feature	Description ^a^
Type of transmission	Person-to-person (21); Foodborne (3); Waterborne (1)
Setting	Nursing homes (12); Youth hostels/Campgrounds (4); Socio-health centers (3); Kindergartens/Preschool centers (3); Schools (2); Hotel (1)
Human norovirus (HuNoV) genotype(s)	GII.17[P17] (5); GI.4[P4] (4); GII.4[P31] (3); GII.4[P16] (2); GI.1[P1] (2); GII.2[P16] (3); GI.6[P11] (2); GII.1[P33] (1); mixed GI.3[P13] + GI.2 (1); mixed GI.4[P4] + GII.4[P16] (1); mixed GI.3[P13] + GII.17[P17] (1)
Duration of outbreak in days: range [median] ^b^	1–25 [9]
Number of affected individuals: range [median]	7–87 [24]
Attack rate (%): average ± standard deviation ^c^	32 ± 20
Days passed between the onset of the outbreak and date of sampling: range [median] ^c^	0–17 [5]

^a^ Numbers in parentheses indicate the number of outbreaks; numbers in brackets indicate median values. ^b^ Information was available for 22 outbreaks. ^c^ Information was available for 24 outbreaks.

**Table 2:** Subjects included in the study.

Link with the Institution	Symptom Status			
	Cases	No Cases	No Data	Total
Users (residents, students, etc.)	237	67	0	304
Workers	71	125	1	197
No Data	2	3	0	5
Total	310	195	1	506

**Table 3:** Prevalence of HuNoV RNA in saliva samples of studied subjects, according to the genotype to which they were exposed.

		Symptomatic Cases		Asymptomatic Exposed Subjects		Total	
Genotype	No. of Outbreaks	No. of Analyzed Samples	No. of Positive Samples (%)	No. of Analyzed Samples	No. of Positive Samples (%)	No. of Analyzed Samples	No. of Positive Samples (%)
**GI.1[P1]**	2	25	0 (0%)	15	0 (0%)	40	0 (0%)
**GI.3[P13]**	1	10	0 (0%)	10	0 (0%)	20	0 (0%)
**GI.4[P4]**	4	25	0 (0%)	20	0 (0%)	45	0 (0%)
**GI.6[P11]**	2	11	0 (0%)	5	0 (0%)	16	0 (0%)
**GI.3[P13] + GI.2**	1	4	0 (0%)	4	0 (0%)	8	0 (0%)
**Total GI**	10	75	0 (0%)	54	0 (0%)	129	0 (0%)
**GII.1[P33]**	1	13	0 (0%)	18	0 (0%)	31	0 (0%)
**GII.2[P16]**	3	23	6 (26.1%)	10	0 (0%)	33	6 (18.2%)
**GII.4[P16]**	4	30	5 (16.7%)	14	2 (14.2%)	44	7 (15.9%)
**GII.4[P31]**	2	17	3 (17.6%)	0	0 (0%)	17	3 (17.6%)
**GII.17[P17]**	6	57	11 (19.3%)	54	3 (5.5%)	111	14 (12.6%)
**Total GII**	16	140	25 (17.9%)	96	5 (5.2%)	236	30 (12.7%)

**Table 4:** Risk factors for occurrence of HuNoV in saliva in GII studied cases (* *p* < 0.05).

Factor		Saliva+	Saliva−	HuNoV Positivity in Saliva	Odds Ratio	CI 95%	*p* Value
Vomiting (*n* = 105)	Yes	16	66	19.5%	0.687	0.233–2.021	0.501
	No	6	17	26.1%			
Nausea (*n* = 90)	Yes	12	28	30.0%	2.633	0.924–7.497	0.074
	No	7	43	14.0%			
Fever (*n* = 94)	Yes	1	17	5.6%	0.176	0.0220–1.416	0.067
	No	19	57	25.0%			
Diarrhea (*n* = 109)	Yes	16	52	23.5%	1.495	0.557–4.013	0.440
	No	7	34	17.1%			
Abdominal pain (*n* = 95)	Yes	11	40	21.6%	1.069	0.397–2.88	0.900
	No	9	35	20.5%			
Consumption of proton pump inhibitors (*n* = 85)	Yes	5	12	29.4%	1.607	0.485–5.322	0.450
	No	14	54	20.6%			
Secretor Status (*n* = 130)	Pos	24	94	20.3%	2.809	0.345–22.830	0.352
	Neg	1	11	8.3%			
Age (*n* = 129)	≥65 years	18	48	27.3%	3.000	1.155–7.791	0.022
	<65 years	7	56	11.1%			
Sex (*n* = 131)	Male	4	30	11.8%	0.482	0.153–1.523	0.216
	Female	21	76	21.6%			

CI: confidence interval.

**Table 5:** Viability propidium monoazide (PMA)xx RTqPCR assay of HuNoV GII present in artificially contaminated saliva and in the saliva of one patient.

Sample	Treatment	PMA	HuNoV RNA Log Genome Copies/mL	Log Reduction *
Artificially contaminated saliva	Untreated	-	6.72 ± 0.01	0.03
		+	6.69 ± 0.01	
	5 min at 95 °C	-	2.91 ± 0.03	0.97
		+	1.94 ± 0.47	
Patient	Untreated	-	3.45	0.30
		+	3.15	

* Reduction in titers obtained between samples before and after viability pretreatment.
